# The Influence of Forefoot Bending Stiffness of Futsal Shoes on Multiple V-Cut Run Performance

**DOI:** 10.3389/fpsyg.2021.625079

**Published:** 2021-06-03

**Authors:** Shariman Ismadi Ismail, Hiroyuki Nunome, Yuji Tamura

**Affiliations:** ^1^Faculty of Sports Science and Recreation, Universiti Teknologi MARA, Shah Alam, Malaysia; ^2^Graduate School of Sports and Health Science, Fukuoka University, Fukuoka, Japan; ^3^Faculty of Sports and Health Science, Fukuoka University, Fukuoka, Japan

**Keywords:** futsal shoe, bending stiffness, sole hardness, running, change of direction

## Abstract

A forefoot bending stiffness (FBS) property of footwear is known to benefit athletes in running performance. To date, the efficacy of bending stiffness on performance is rather unknown from the perspective of futsal shoes. This study investigates the influence of bending stiffness property of three commercial futsal shoes on change of direction run resultant performance. Nineteen university level athletes participated in the human performance test (multiple V-cut change of direction run) on a hardwood flooring facility using three pairs of futsal shoes (i.e., S1, S2, and S3) with different models but similar in outsole material (S1—mass: 311 g, heel-to-toe drop: 10 mm, friction coefficient, 1.25; S2—mass: 232 g, heel-to-toe drop: 8 mm, friction coefficient: 1.34; and S3—mass: 276 g, heel-to-toe drop: 7 mm, friction coefficient: 1.30). The FBS properties for each shoe were mechanically measured. Results from the analysis of variance indicated that there was a significant difference of FBS value among the three shoes (S1: 0.32 Nm/deg., S2: 0.26 Nm/deg., and S3: 0.36 Nm/deg.) [*F*_(2,8)_ = 28.50 (*p* < 0.001)]. Shoes with relatively higher shoe-playing surface friction coefficient (S2 and S3) had significant impact on the V-cut performance (*p* < 0.05) when compared with the shoe with lower friction coefficient (S1). In contrast to the literature, the shoe with the lowest FBS (S2) did not suffer any detriments on the resultant performance in the test conducted. These findings suggested that there could be other performance limiting factors, such as the friction coefficient, rather than FBS that have greater influence on the test outcomes.

## Introduction

Similar to soccer, futsal is a sport that demands intermittent high-intensity activity which is based not only on aerobic but also on anaerobic capacity (Bangsbo et al., 1991; Barbero-Alvarez et al., 2008). In a futsal game, players received constant pressure from the opponent players throughout the match, where a 1 vs. 1 situation is common (Vaeyens et al., 2007). Thus, futsal players need to frequently perform a change of direction motion, i.e., turning and other acceleration–deceleration type of movements (Vaeyens et al., 2007). Therefore, the aspect of agility and change of direction capability is crucial in futsal game. Previous studies that compared futsal and soccer players found no significant difference between them on agility and change of direction performances (Milanović et al., 2011; Kartal, 2016). While many studies have focused on the aspect of shoes on soccer performance, there are still only few studies that focus on the influence of shoes on the performance of players in futsal (Kulessa et al., 2017).

Appropriate shoe selection would contribute to improve the performance of players and help them reduce injury risks based on the sport-specific functionality (Lake, 2000). A lower-extremity injury is common in soccer, and the previous study has shown that shoe property can influence biomechanical variables that are related to lower-extremity injuries such as peak dorsiflexion and knee flexion angle (Butler et al., 2014). Among many aspects of a shoe, the importance of bending stiffness has been highlighted in many previous studies where adequate shoe bending stiffness is known to benefit athletes in the running performance (Stefanyshyn and Fusco, 2004; Tinoco et al., 2010; Worobets and Wannop, 2015). It has been known that stiffer shoe configuration helps in reducing the ground reaction force during the push-off phase in running (Willwacher et al., 2014). In addition, higher shoe bending stiffness has also been reported to improve vertical impulse and vertical jump height in a countermovement jump (Roy and Stefanyshyn, 2002). To date, there are several studies on the efficacy of forefoot bending stiffness (FBS) on the running performance for athletic shoes (Stefanyshyn and Fusco, 2004), volleyball shoes (Tinoco et al., 2010), and basketball shoes (Worobets and Wannop, 2015) but none on futsal shoes (Kulessa et al., 2017). Each of the above-mentioned studies has reported positive results when using shoes with higher bending stiffness properties for each respective sport. This feature of futsal shoes also warrants investigation.

In this study, we aimed to generate new information with regards to the commercially available futsal shoes. We also aimed to investigate bending stiffness efficacy on the functionality aspects of futsal shoe. Therefore, the purpose of this case study is to examine futsal shoe bending stiffness influence on multiple V-cut run performance. It was hypothesized that shoe bending stiffness would have a substantial impact on the test outcomes.

## Materials and Methods

### Participants

Nineteen experienced male university level soccer players who regularly participated in competitive national level soccer tournaments (age: 20.2 ± 1.1 years old, body mass: 66.8 ± 6.7 kg, height: 174 ± 5 cm, and soccer experience: 13.6 ± 2 years) were recruited for the multiple V-cut test. All participants were free from any lower limb injuries and active in university level competitions when they participated in this study during the off-season period. All participants provided written informed consent prior to participation in accordance with the research ethical approval obtained from the institutional research ethics committee.

### Footwear

Three different models of shoes were selected for this study. The properties and features of each shoe are described in Table 1.

**Table 1:** Properties and features of shoes.

**Shoe**	**S1 (Puma Invicto II) 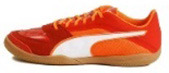 **	**S2 (Mizuno Monarcida Sala) 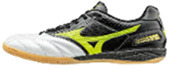 **	**S3 (Mizuno Monarcida FS) 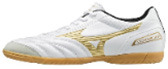 **
Shoe mass (g)	311	232	276
Heel-to-toe drop (mm)	10	8	7
Hardness of outsole [Shore HA (°)]	57	60	60
Available friction coefficient (AFC^*^)	1.25	1.34	1.30

* *Data based on the previous study (Ismail et al., 2020), reproduced with permission from the publisher (license number: 4937400524403)*.

The selection criteria for the shoes are as follows: (1) categorized as an indoor soccer/futsal shoes, (2) did not possess midsole construction, (3) possessed similar outsole material and hardness, (4) similar heel-to-toe drop value, (5) available friction coefficient (AFC) differences within ±20%, and (6) mass differences within ±20% and differences below 300 g (Nigg and Enders, 2013; Worobets and Wannop, 2015). These criteria were prerequisite to ensure that all the selected shoes possessed no obvious difference in terms of the shoe construction, and any differences would not provide any obvious advantages/disadvantages during the test conducted in this study.

### Change of Direction Run Test and Experimental Procedure

**Figure 1:**
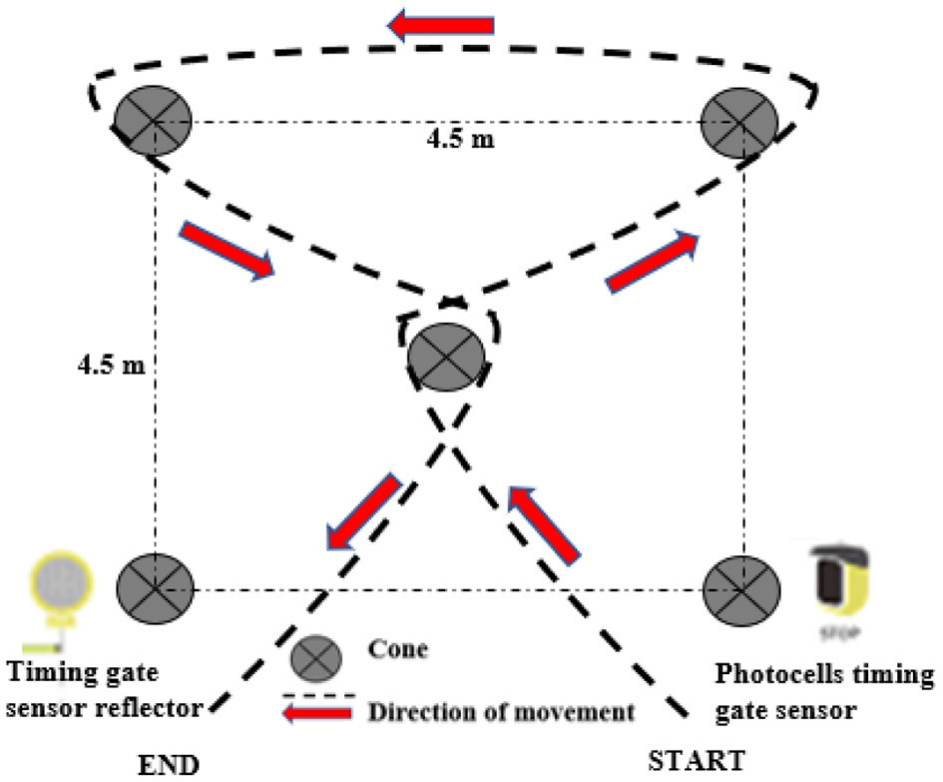
Multiple V-cut pylon course functional test.

A cutting course similar to that of the study by Worobets and Wannop (2015) was set in a hardwood indoor flooring facility. Before testing, each participant performed an adequate warm-up that was similar to his usual preparation for a match. Each participant was then asked to maximally complete this cutting course consisting of two 90° cut and 135° V-cut maneuvers within a 4.5 m × 4.5 m area (Figure 1) using the three types of shoes with different sizes. During the test, the order of shoes was randomized (no two consecutive trials using the same shoe). The test was repeated with enough intervals (to avoid the effect of fatigue) between trials to obtain three successful trials (no slipping and colliding with cone during trial) for all the three shoes. A photocell timing gate system (Witty System, Microgate, Italy) was utilized to record the resultant time of all the trials. The distance between the participant front foot and the photocell timing gate sensor during the beginning of the trial was fixed at 0.5 m. All the tests were performed on hardwood indoor flooring facilities.

### Shoe Forefoot Bending Stiffness Test

The FBS of each shoe was measured using a similar method described in the study by Worobets and Wannop (2015). Each shoe was applied with forefoot bending forces ranging between 2 and 18 Nm in 6–9 stages until all shoes reach a maximum bending angle of more than 40°. All data obtained from these trials were plotted on a bending force–angle graph as the bending force was the independent variable, and a regression line was fit at least to a minimum of six data points. The bending stiffness of each shoe was measured three times, and the mean values were computed as the representing values.

### Data and Statistical Analysis

In this study, the results obtained for all the three shoes were compared to observe any differences in terms of the bending stiffness properties and resultant running performances during the change of direction run test.

The statistical analyses were performed using an open-source statistical software, PSPP (GNU project, version 1.0.1). A comparative analysis across the three shoes was conducted with one-way ANOVA repeated measures, and Bonferroni *post-hoc* analysis was applied when required. The statistical significance level was set at *p* < 0.05 for all the analyses.

## Results

### Forefoot Bending Stiffness

The FBS for each shoe and the comparative analysis across the three shoes (i.e., one-way ANOVA repeated measures with Bonferroni *post-hoc* analysis) are shown in Table 2. It was identified that there was a significant difference between the S2 (0.26 Nm/deg.) and the other two shoes [S1: 0.32 Nm/deg. (*p* = 0.01) and S3: 0.36 Nm/deg. (*p* < 0.001)].

**Table 2:** The mean forefoot bending stiffness of each shoe.

**Shoe**	**Forefoot bending stiffness**
	**Mean ± (SD) [Nm/degree]**
S1	0.32 ± 0.02^*^ (*p < 0.05)
S2	0.026 ± 0.01 (*p < 0.05; *p* < 0.001)
S3	0.36 ± 0.02 (*p* < 0.001)
ANOVA remarks	*F*_(2,8)_ = 28.50 (*p* < 0.001)

*Post-hoc pairwise comparisons*.

* *Significantly different between S1 and S2*.

*Significantly different between S2 and S3*.

*All statistical significance level at p < 0.05*.

### Multiple V-Cut Performance

**Figure 2:**
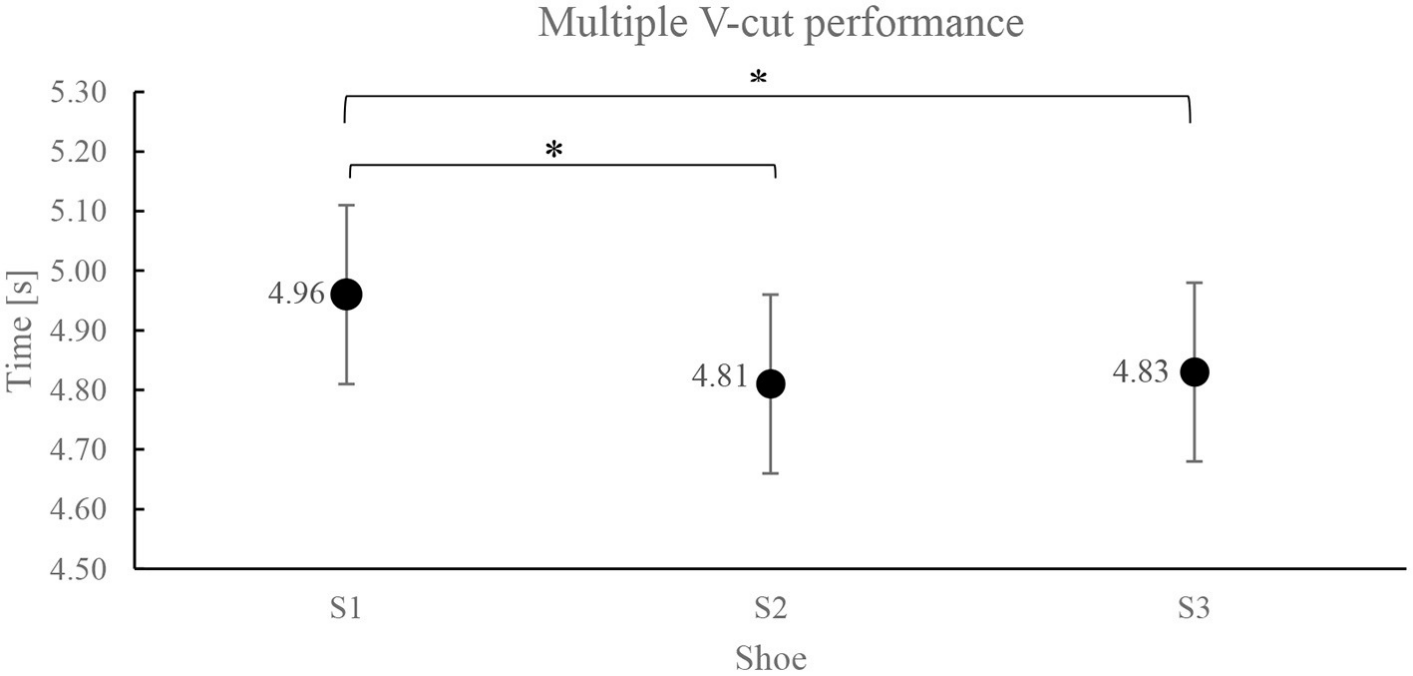
Mean value (±SD) of multiple V-cut performance (*ANOVA: *p* < 0.05).

The results obtained from the multiple V-cut run performance test are shown in Figure 2. As shown in the figure, there are significant differences among the three shoes for the multiple V-cut performance test [*F*_(2,170)_ = 4.60 (*p* = 0.01)] where the participants performed significantly faster when they used the S2 (4.81 ± 0.3 s: *post-hoc p* = 0.02) and S3 shoes (4.83 ± 0.3 s: *post-hoc p* = 0.03) than S1 (4.96 ± 0.3 s) while no significant difference was found between S2 and S3 shoes.

## Discussion

This study aimed to verify FBS efficacy on the resultant performance in change of direction run task. We hypothesized that different FBS properties would have a substantial impact on the outcomes of the functional performance tests. It was demonstrated surprisingly that FBS is considered to have no substantial impact on the performance tests conducted in this study. Thus, these findings most likely rejected our hypothesis that bending stiffness for the tested shoes in this study would have a substantial impact on multiple V-cut run resultant performance.

### Forefoot Bending Stiffness

For the FBS, the S1 shoe with a softer outsole material (57 HA) has shown to possess significantly higher bending stiffness (S1: 0.32 Nm/deg., S2: 0.26 Nm/deg.) when compared with the S2 shoe (60 HA). In addition, we also found a significant difference between S2 and S3 shoes (Table 2), where both of them have identical outsole material hardness (60 HA). This finding demonstrated that the hardness of outsole material is not a primary factor to determine the FBS of a shoe. It can be assumed that the stiffness could potentially be modulated by the straight-line groove aspect of the outsoles (Lam et al., 2019). The S1 and S3 shoes do not have aggressive medio-lateral straight-line grooves as S2 shoe. This simplex outsole feature could potentially influence the bending stiffness property of S2. As intended, this feature likely affects its relatively lower bending stiffness when compared with other shoes. The results from this study have demonstrated that the outsole geometrical aspects could have a substantial influence on the FBS of a shoe. In addition, the upper sole material and construction of each shoe could also dictate the FBS properties. Another possible explanation on the lack of influence of the FBS of a shoe on the functional test results is that the FBS of all the shoes selected in this study may be much smaller than the FBS of the participants. If the FBS of a human is larger than the bending stiffness of a shoe, then the total FBS will be dominated by the human foot, thus limiting the influence of the FBS of shoes during the functional test. A similar finding was reported in the literature when it was found that if the FBS of a human is larger than the bending stiffness of the running shoes, then any variations in the bending stiffness of the shoes are unlikely to have a significant influence on running performance (Oleson et al., 2005).

### Multiple V-Cut Performance

It was generally accepted that higher FBS is known to benefit athletes in running performance (Stefanyshyn and Fusco, 2004; Tinoco et al., 2010; Worobets and Wannop, 2015; Day and Hahn, 2020). The studies by Worobets and Wannop (2015) and Day and Hahn (2020) have reported the FBS values (0.10–0.29 Nm/deg. and 0.22–0.33 Nm/deg., respectively) by using a similar method performed in this study. Both studies reported that higher FBS significantly improved running performances. In this study, however, the differences in FBS of the tested shoes do not seem to play a dominant role for running performance because the shoe with the lowest FBS (S2) did not suffer any detriments on resultant performance in change of direction results. The contradicting results found in this study may be due to the fact that these two previous studies (Worobets and Wannop, 2015; Day and Hahn, 2020) applied systematic ways to solely alter the shoe bending stiffness. Under this circumstance, only the FBS property of the tested shoes was altered while retaining the shoe property identical. On the contrary, in this study, an attempt was made to compare commercially available shoes across different models but with similar features and construction. Based on this evidence, it can be speculated that that there could be other performance limiting factors rather than FBS which have influenced the test outcomes during the multiple V-cut change of direction.

### Influence of Shoe Mass

The previous study has verified that increasing the shoe mass would predictably reduce running performance from the energetic point of view (Hoogkamer et al., 2016). From the perspective of sprint and cutting performances, the effect of shoe mass seems to have some threshold. The study by Nigg and Enders (2013) on sports footwear found that only shoe mass becomes an important factor for the weight difference of above 300 g in running performance. This finding has been supported by other similar studies. Recent study by Köse (2018) verified that the weight difference of 285 g between two shoes has shown a significant effect on 10-m sprint performance. In another study on basketball shoe, it was reported that the shoe within 20% of weight difference did not significantly influence the sprint and cutting performances (Worobets and Wannop, 2015). Since the three shoes used in this study are within 20% of weight differences (below 285 g of weight differences), it was concluded that the influence of shoe mass is less than small or non-existent due to small weight differences.

### Influence of Heel-to-toe Drop

The three shoes selected for this study possess relatively small heel-to-toe drop differences (7–10 mm) when compared with one another. While it could not be identified precisely how this small difference could influence the study outcomes, the only possibility that exist is the shoe with higher heel-to-toe drop could possibly offer a slight extra cushioning feature to the shoe. However, the study by Lam et al. (2017) identified that cushioning provides no advantage to the horizontal ground reaction force component that is crucial during short-exertion, high-intensity movement such as short-distance sprint and change of direction tasks. Therefore, it can be suggested that the influence of heel-to-toe drop difference was minimal in this study.

### Influence of Available Friction Coefficient

The three shoes selected in this study were also selected in other previous study (Ismail et al., 2020), where the available traction (the AFC) of all the shoes were mechanically measured. The influence of AFC on change of direction and perceived traction performances was observed in the study by Ismail et al. (2020). It was reported that AFC possessed substantial influence on the change of direction and perceived traction performances. Similarly, as reported in this study, Ismail et al. (2020) reported that participants have performed significantly better when using S2 (AFC: 1.34) and S3 (AFC: 1.30) shoes as compared with S1 shoe (AFC: 1.25). Thus, it was suggested that differences on the AFC component between the shoes of S1 and S2 as well as S1 and S3 could potentially influence the outcomes of the study. Currently, there is no existing study that has compared the influence of both AFC and FBS on change of direction performance. Therefore, it is still difficult to establish a clear conclusion, but as observed in this study and previous study (Ismail et al., 2020) it can be speculated that AFC could potentially possess a much dominant influence on change of direction performance as compared with FBS.

### Study limitation

This study focused only on the commercially available futsal shoes to provide more practical, user-friendly information. Although careful selection criteria to pick three different futsal shoes representing similar features were made, several factors, including outsole groove patterns and shoe upper materials and construction, were not systematically controlled. Therefore, the results observed in this study might not be generalized for all types of futsal shoes. More testing on various futsal shoe model is warranted. In addition, we tested the shoe with only one playing surface (hardwood). The tests on different types of futsal playing surfaces (e.g., vinyl, plastic-based, or rubberized material) should be warranted in future studies.

## Conclusion

In this study, it was demonstrated that FBS may not have systematic influence on resultant performance of the multiple V-cut run. Other possible factors such as mass and heel-to-toe drop properties of shoes were also considered as not having substantial influence on the performance tested in this study. This is likely due to the fact that the property of tested shoes, namely the AFC, possessed the performance limiting factors, namely, mass or heel-to-toe drop properties, rather than FBS. The AFC of a futsal shoe could possess a high dominant effect on change of direction performance rather than FBS.

## Data Availability Statement

The raw data supporting the conclusions of this article will be made available by the authors, without undue reservation.

## Ethics Statement

The studies involving human participants were reviewed and approved by Fukuoka University, Fukuoka, Japan. The patients/participants provided their written informed consent to participate in this study.

## Author Contributions

SI has conducted the research conceptualization and data collection, and written the main section of the manuscript. HN and YT have substantially contributed to the supervision, design of the work, and critical review and discussion of the manuscript. All authors contributed to the article and approved the submitted version.

## Conflict of Interest

The authors declare that this study received funding from Mizuno Corporation. Mizuno Corporation had the following involvement with the study: sponsoring the footwear used in the study. Mizuno Corporation was not involved in the study design, collection, analysis, interpretation of data, the writing of this article, or the decision to submit it for publication.
